# Dyes in Liquid Crystals: Experimental and Computational Studies of a Guest–Host System Based on a Combined DFT and MD Approach

**DOI:** 10.1002/chem.201406372

**Published:** 2015-06-01

**Authors:** Mark T Sims, Laurence C Abbott, Stephen J Cowling, John W Goodby, John N Moore

**Affiliations:** [a] Department of Chemistry, The University of YorkHeslington, York YO10 5DD (UK), Fax: (+44) 1904-322516

**Keywords:** density functional calculations, dyes/pigments, liquid crystals, molecular dynamics, UV/Vis spectroscopy

## Abstract

Practical applications of guest–host liquid crystal systems are critically dependent on the alignment of the guest species within the liquid crystal host. UV/Vis absorption spectroscopy shows that the 1,5-dihydroxy-2,6-bis-(4-propylphenyl)-9,10-anthraquinone dye aligns within the E7 nematic host, giving an experimental dichroic ratio of 9.40 and dye order parameter of 0.74. This alignment was modelled by using a combination of density functional theory (DFT) and molecular dynamics (MD) computational approaches that do not require the input of experimental data. Time-dependent DFT calculations show that the electronic transition dipole moment is highly aligned with the long molecular axis of the dye. Fully atomistic MD simulations show that the long axis of the dye is less highly aligned within the E7 host, indicating that this contribution limits the overall dye alignment and, thereby, the potential practical applications of this particular system. Importantly, this study demonstrates an experimental and combined DFT and MD computational approach that may be applied generally to guest–host systems, providing a potential route to their rational design.

## Introduction

**Figure 1:**
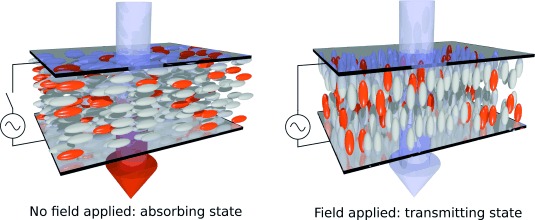
Schematic diagram of a guest–host device in the absorbing/coloured state without an applied electric field (left) and in the transmitting/colourless state when an electric field is applied (right), with incident white light. The dye molecules are shown in orange and molecules of a nematic host are shown in grey.

The potential for using dyes in liquid crystal devices arose from the first reported observation of dye alignment in a nematic host,[1] and numerous modes of guest–host device operation have been suggested subsequently.[2,3] These devices are based on the principle that the guest chromophore aligns with the liquid crystal host, enabling the application of an electric field to switch the orientation of the liquid crystal matrix between that of an absorbing (coloured) state and a transmitting (colourless) state, as shown schematically in Figure 1, because the absorption transition is aligned along a specific axis within the dye. Such guest–host devices do not necessarily require polarizers or colour filters because the absorption properties are defined by the guest dye molecules and their alignment; hence, they may provide more robust devices with higher optical efficiencies and lower power consumption than some more conventional liquid crystal displays (LCDs), including the potential for colour displays operating in ambient light-scattering mode without the need for back-lighting.[3,4]

For a guest–host system to be suitable for a display application, it must fulfil a number of criteria. Of primary importance for the visual properties of a device are the colour and absorption coefficient of the dye, the solubility of the dye in the host, and the contrast ratio between the absorbing and transmitting states, which is determined by the alignment of the dye within the guest–host mixture. In addition, for a device to be useful practically, the system must be thermally, photochemically and electrochemically stable to provide a suitable operational lifetime that may be several years for typical display applications. Therefore, the choice of both the dye and the liquid crystal host, as well as their mutual compatibility, are crucial in designing a successful device.

Azo dyes were initially suggested for use in guest–host displays because of their elongated, rod-like molecular shape aiding alignment with a liquid crystal host that typically comprises rod-like molecules.[5] A range of colours is also readily achievable with the well-established synthetic chemistry of azo dyes, but their limited photochemical and electrochemical stability can give an inherent barrier to their use in practical display devices.[6] Anthraquinone dyes have been suggested as alternative guests because they are typically more stable than azo dyes, but their molecular shapes are usually less rod-like and, consequently, their alignment within the host tends to be poorer than that of azo dyes.[7] Good alignment in liquid crystal hosts has been obtained for some anthraquinone dyes, but it has proved challenging to obtain a range of colours with high absorption coefficients.[8,9] Many other classes of dyes have also been synthesised for possible use in guest–host systems including tetrazines,[10] naphthalenes,[11] perylenes[12] and acenequinones,[13] illustrating the wide range of compounds that offer potential for these applications, and research into new dyes for display devices is ongoing.[14]

The choice of host for these applications is also important, and many different mixtures with dyes have been tested, including nematic,[14,15] chiral nematic[16,17] and smectic[18,19] systems. Of these hosts, nematic systems are the simplest and the most widely studied. Chiral nematic systems potentially offer improved optical efficiency, and smectic A hosts can provide the additional advantage of bistability,[20] offering the prospect of devices with lower power consumption than other guest–host devices.

Although the main focus has been on their applications in display devices, dyes in liquid crystal hosts have also been proposed as the basis for materials giving diverse applications, including optical storage devices,[21] “smart” windows,[22] switchable waveguides,[23] high-contrast polarizers[24] and polarized electroluminescent light sources.[25] Due to the number of criteria that guest–host systems must fulfil for their successful use in practical display and other devices, an approach based on the rational design of both guest and host molecules is desirable. For most guest–host applications of liquid crystals, the effectiveness of the system is crucially dependent on the absorption of aligned guest molecules within the device, such that a knowledge and understanding of the properties that define this behaviour are key requirements to underpin an approach towards their rational design.

An understanding of the electronic absorption transitions giving rise to the colours of dyes can now be aided greatly by time-dependent density functional theory (TD-DFT) calculations, typically on molecules isolated in the gas phase or in a solvent field. These calculations can give good matches between experimental and calculated data, and they provide information on the orbital nature, energies and strengths of the transitions. Examples of such studies include those on azo[26,27] and anthraquinone[28,29] classes, with reports often focusing on these systems in the context of textile fibre dyeing and related industrial applications.

Molecular dynamics (MD) simulations have been widely used to study liquid crystals, providing insight into their behaviour and giving successful comparisons with various experimental properties, including alignment, transition temperatures and rotational viscosities. MD approaches have included coarse-grain methods, in which the molecules are treated as single geometric volumes,[30] united-atom approaches, in which groups of atoms are combined,[31,32] and fully atomistic simulations, in which all of the atoms are treated explicitly.[33] A recent report described the use of MD simulations to explore the alignment of small rigid solute guests in a nematic host,[34] although guest–host simulations appear to have received relatively little attention to date.

**Figure 2:**
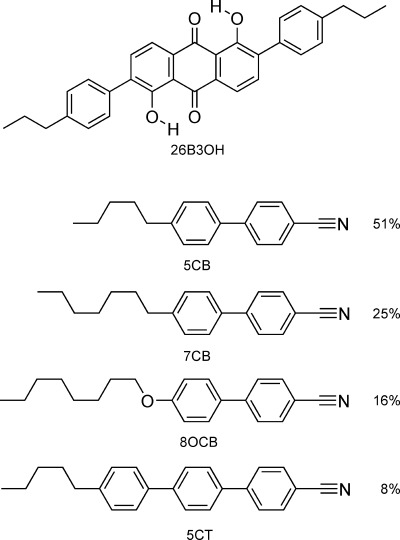
Structures of the anthraquinone dye (26B3OH) and the components of the E7 nematic mixture, along with their abbreviations and the E7 composition by wt %.

Here, we present a new general approach to modelling the optical and alignment properties of dyes in liquid crystal hosts that is based on a combined computational method using DFT calculations and fully atomistic MD simulations, allied with experimental data on the system being modelled. We report studies on a recently synthesised 2,6-disubstituted anthraquinone dye (26B3OH),[35] shown in Figure 2, which is of particular interest as a liquid crystal guest dye because of its relatively rod-like molecular shape. The host used in these studies, both experimental and computational, was the nematic mixture E7, as given in Figure 2, which has been the subject of many experimental and computational studies.

## Results and Discussion

### Experimental spectra of an aligned guest–host system

**Figure 3:**
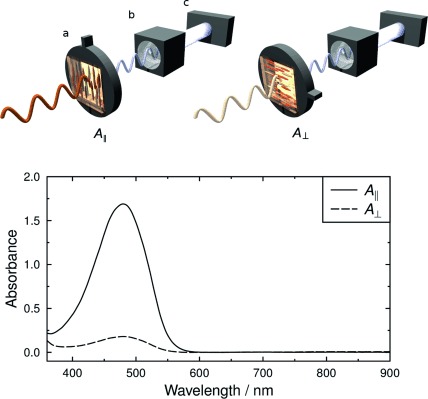
Experimental UV/Vis absorption spectra of an aligned guest–host sample of 26B3OH at approximately 1.5 wt % in E7 at 300 K with the sample alignment set parallel (II) and perpendicular (⊥) to the polarizer, as shown in the schematic diagram above (a=aligned guest–host sample; b=polarizer; c=white light source).

The experimental UV/Vis absorption spectra of an aligned sample of 26B3OH in E7 obtained by using linearly polarized light are shown in Figure 3. The sample gave a polarized absorption band at approximately 480 nm, and exhibited an orange/red colour consistent with this band position.

The alignment of the dye molecules can be quantified by using the experimental dichroic ratio of the dye, *R*, defined as the ratio of the absorbances of the dye in the aligned sample orientated parallel (*A*_II_) and perpendicular (*A*_⊥_) to the incident polarized light (Figure 3 schematic). These values may be used in Equation (1)[6,36] to obtain the experimental order parameter of the dye *S*_exptl_, for which a value of 1 indicates that the dye molecules are fully aligned, and a sample in which the dye molecules exhibit no alignment gives a value of 0.

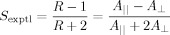
(1)

The aligned sample of 26B3OH in E7 gave a dichroic ratio of *R*=9.40, measured across the range of the visible absorption band, from which an experimental order parameter of *S*_exptl_=0.74 was obtained. These values are relatively high for an anthraquinone dye, consistent with the relatively rod-like shape of the 2,6-disubstituted structure of 26B3OH.

### Contributions to the experimental alignment and order parameter

In a nematic phase, the molecules align along a preferred axis termed the director and defined by a unit vector **n**. The alignment can be quantified by the order parameter *S*_*θ*_, given by Equation (2), in which *θ* is the angle between the director and the long axis of an individual molecule, *P*_2_ is the second Legendre polynomial, and the brackets <..> denote an ensemble average over a number of molecules and/or time.

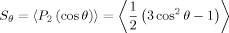
(2)

**Figure 4:**
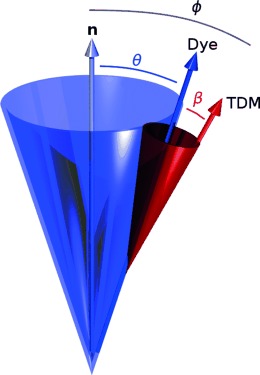
Schematic representation of the relative orientations of the director n for a nematic liquid crystal system, the long axis of a guest dye molecule (blue), and the TDM of the dye (red), with angles defined.

Guest molecules that align with a liquid crystal host adopt the same director **n**. In the case of a guest dye, the experimental measurement of the alignment obtained from a UV/Vis dichroic ratio gives the order parameter of the associated electronic transition dipole moment (TDM) of the dye, rather than that of the long axis of the dye. A schematic representation of this TDM orientation is shown in Figure 4, in which *ϕ* is the angle between the director and the electronic TDM; this alignment results in the order parameter of the dye *S*_*ϕ*_, given by Equation (3), which equates here to the experimental order parameter *S*_exptl_, given by Equation (1).

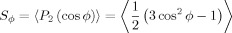
(3)

As shown in Figure 4, the alignment of the TDM of a uniaxial dye molecule can be considered to arise from two contributions: the alignment of the long axis of the dye molecule with the director, defined by angle *θ*, and the alignment of the TDM with the long axis of the dye molecule, defined by angle *β*. These contributions give rise to associated order parameters *S*_*θ*_ and *S*_*β*_, respectively, as defined in Equation (4), the product of which equates to *S*_*ϕ*_ and, hence, to the experimental value *S*_exptl_.[6,36]

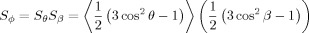
(4)

The ability to consider the contributions to the experimental order parameter in terms of a component from the molecular alignment of the guest within the host and a separate component from the alignment of the transition dipole moment within the dye is very useful. From the perspective of materials design, it provides a simplification of the problem into two features, and computationally it enables the two contributions to be calculated independently of each other. For this model to be used, the long molecular axis of the dye must be defined quantitatively, and in the work reported here it is taken to be the axis of minimum moment of inertia, as has been suggested and used in previous studies.[33,37,38]

### Transition dipole moment alignment within the dye

**Figure 5:**
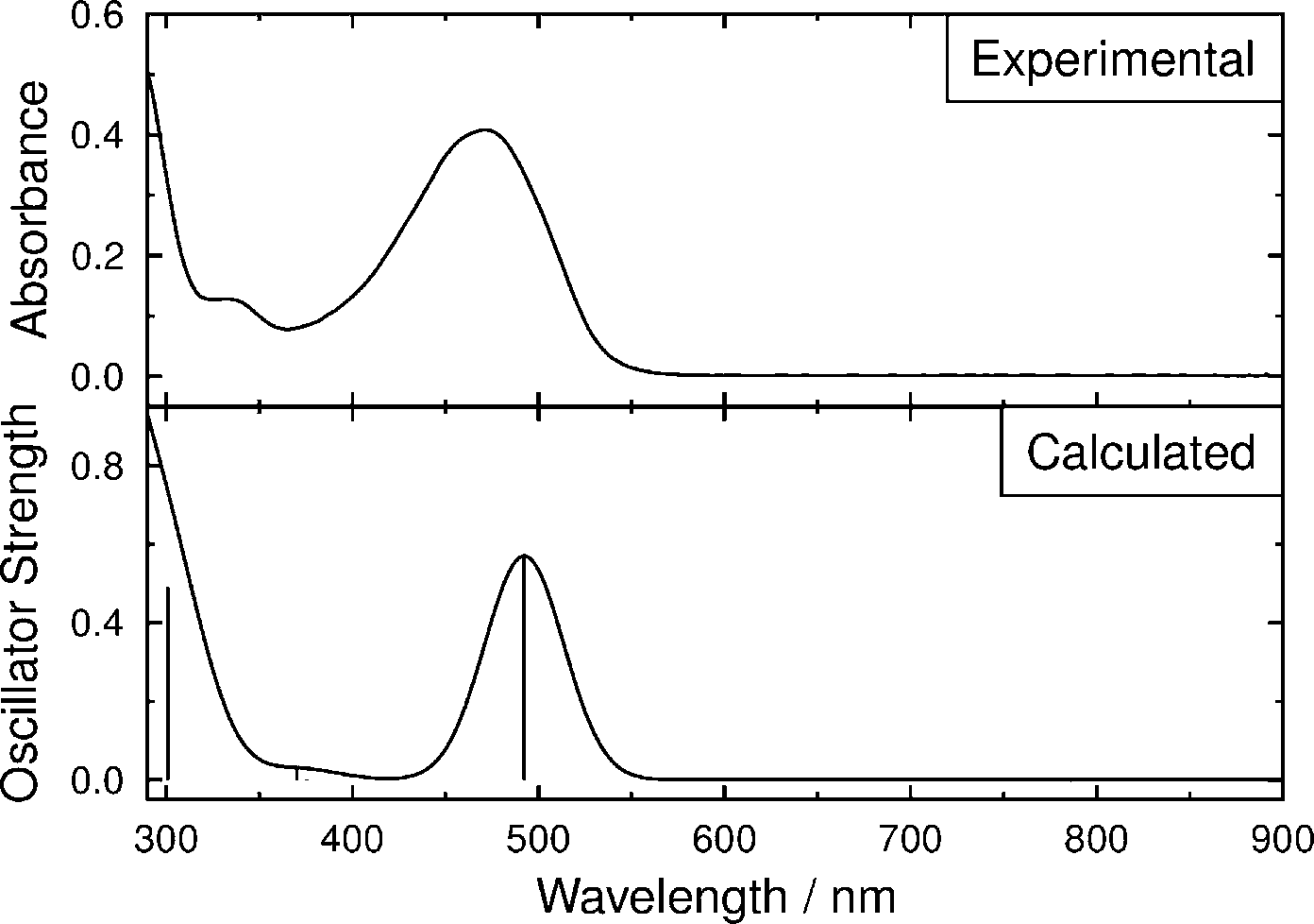
Experimental UV/Vis absorption spectrum of 26B3OH at 2.0×10^−4^ mol dm^−3^ in *p*-xylene (top), and simulated UV/Vis absorption spectrum of 26B3OH with vertical lines showing the wavelengths and oscillator strengths of the transitions calculated at the B3LYP/6-31G(d) level for the isolated molecule (bottom).

The experimental UV/Vis absorption spectrum of an isotropic sample of 26B3OH in *p*-xylene solution is shown in Figure 5. The absorption maximum of the visible band occurs at *λ*_max_=471 nm, resulting in the orange/red colour of the dye. The absorption coefficient at this wavelength was found to be *ε*_max_=2.04×10^4^ dm^3^ mol^−1^ cm^−1^, and integration across this absorption band gives an experimental oscillator strength of *f*_exptl_= (*k*/*n*)∫*ε*($\tilde \nu $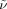)d$\tilde \nu $
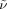
≈0.28,[39] by using values of the constant *k*=4.32×10^−9^ cm mol dm^−3^ and the refractive index *n*=1.50.

**Figure 6:**
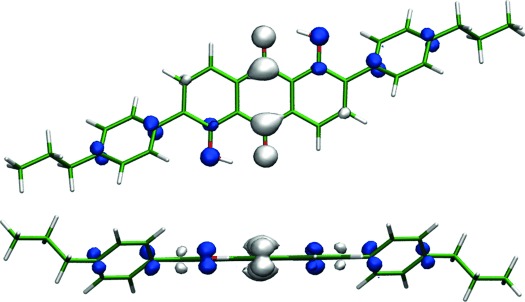
Optimised structure of 26B3OH calculated at the B3LYP/6-31G(d) level, with calculated changes in electron density on excitation from the ground state to the first excited singlet state shown perpendicular to (top) and along (bottom) the anthraquinone plane; blue and white regions represent a decrease and increase in electron density, respectively.

DFT calculations were used to provide a computational comparison with the experimental data. The DFT optimised structure of 26B3OH in the gas phase exhibits a planar anthraquinone core, with the hydroxyl groups lying in the same plane and the phenyl substituents rotated by 41° out of this plane, as shown in Figure 6. TD-DFT calculations on this optimised structure gave a single allowed electronic absorption transition in the visible region at 492 nm, with a high oscillator strength of 0.57 consistent with a strongly allowed transition, and with other allowed transitions occurring in the UV region at <380 nm. A simulated UV/Vis absorption spectrum based on these calculated transitions is shown in Figure 5, along with the experimental data. The calculated orbital contributions indicate that the allowed visible transition is a simple HOMO–LUMO transition, and the calculated changes in electron density shown in Figure 6 indicate that it involves charge transfer from hydroxyl and phenyl donor substituents to acceptor quinone carbonyl groups, which is consistent with the nature of similar transitions reported for related anthraquinones.[29]

**Figure 7:**
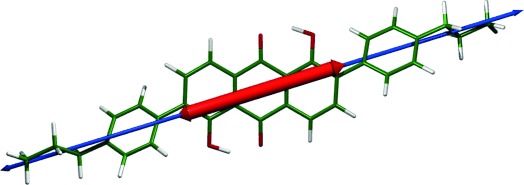
Optimised structure of 26B3OH calculated at the B3LYP/6-31G(d) level, with arrows showing the calculated orientations of the TDM for its visible absorption transition (wide red line) and the long molecular axis (narrow blue line) calculated here as the minimum moment of inertia axis.

The TD-DFT calculations also provide the orientation of the TDM for each transition. Figure 7 shows the optimised structure of the dye overlaid with the orientation of the calculated TDM for the allowed visible transition (shown in red), along with the orientation of the long molecular axis (shown in blue), calculated here as the minimum moment of inertia axis. These two vectors both lie essentially in the anthraquinone plane, and they make an angle of *β*=1.7°, from which the definition in Equation (4) gives an order parameter of *S*_*β*_=0.999 arising from their relative alignment. These values indicate that the TDM for the visible transition of this dye, which gives rise to its colour, is highly aligned with the long molecular axis of the dye. Hence, it is appropriate to consider the alignment of the TDM of the dye within the host to be uniaxial in this context.

### Molecular alignment within a guest–host system

An understanding of the alignment of dye molecules within a host requires the guest–host system to be considered as a whole. Here, we have used fully atomistic MD simulations to model the molecular alignment in this system.

Prior to simulating the guest–host mixture, we carried out fully atomistic MD simulations on 256 molecules of the host E7 mixture alone, in accordance with the methods used in a fully atomistic study of E7 detailed in the literature.[33] Two separate simulations of E7 alone were run, each for 200 ns: one simulation used an isotropic starting geometry, in which the molecules were randomly oriented; and the other used a pseudo-nematic starting geometry, in which the molecules were aligned.[33] The order parameter *S*_*θ*_ of the E7 mixture was calculated as the largest eigenvalue of the ordering tensor, *Q*_*αβ*_, and the director **n** as the associated eigenvector,[33,40] as given by Equation (5), in which *N* is the number of molecules, *δ* is the Kronecker delta, *j* represents the molecule number in the simulation, *α* and *β* represent the Cartesian *x*, *y* and *z* axes, and *a* represents the component of the long molecular axis vector. The long molecular axis was defined as the axis of minimum moment of inertia, which was calculated for each component molecule of E7. These calculations were carried out for the whole ensemble of molecules at each time interval in each simulation data set.

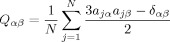
(5)

Our simulations of E7 alone essentially replicate those in the report[33] that first demonstrated the approach we have adopted here; the Supporting Information gives plots of the order parameters (Figure S1) and densities (Figure S2) versus time, and the dihedral angle distribution functions (Figure S3) obtained. The simulation from an isotropic starting geometry evolved to a nematic phase by approximately 75 ns, and averaging over 75–200 ns (the end of the run) gave an order parameter of *S*_*θ*_=0.885; the pseudo-nematic starting geometry enabled averaging over a longer range of 30–200 ns and gave a similar value of *S*_*θ*_=0.877. These values are comparable to those of 0.81 and 0.83, respectively, reported from the final stages of slightly shorter runs carried out by using the same two approaches with a different MD package.[33]

Although the order parameter of E7 simulated here is significantly higher than the experimental values of *S*=0.64 estimated here and *S*=0.65 reported in the literature[33] (see the Supporting Information), the overestimation is consistent with other fully atomistic MD studies of cyanobiphenyl mixtures.[33,41] A key reason for the overestimation is probably the use of a force field in which the non-bonded interactions, and particularly the van der Waals interactions modelled by Lennard-Jones potentials, are not optimised for this type of system, as discussed in the literature;[32,33,41] the development of force fields that are tuned specifically for liquid crystal systems is a very important area of research,[32,34] but we have used the default Lennard-Jones parameters of the OPLS force field here because our aim was to carry out a computational study that did not require parameterisation based on experimental data from the system under study. Another contribution to the overestimation may arise from using the minimum moment of inertia axes to calculate the order parameter; and redefining the internal director **n** of the ensemble at every time interval rather than using an external reference frame inherently maximises the order parameter calculated for the system. An additional simulation carried out with 3872 rather than 256 molecules of E7 showed that increasing the size of the system did not significantly affect the calculated order parameter, as described in the Supporting Information (Figure S4).

**Figure 8:**
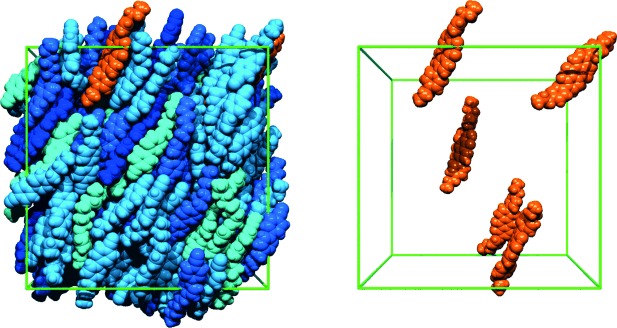
The final geometry of the MD simulation of the guest–host system run from an isotropic starting geometry, showing both dye and E7 molecules (left) and dye molecules only (right); the 26B3OH dye molecules are shown in orange, and the component molecules of E7 are shown in four different shades of blue. The boundaries of the simulation box are shown in green.

A guest–host simulation was run by using the same general conditions as those used for the E7 mixture alone, and from an isotropic starting point. The simulation consisted of 400 molecules of E7 with five dye molecules randomly distributed amongst them; this composition was chosen as a balance between generating sufficient data for meaningful analysis, providing a dye concentration of 2.06 wt %, which is comparable to that of 1.5 wt % for the aligned experimental sample, and giving a reasonable computational time. The simulation was run for 500 ns, which we found was necessary to ensure that each of the five dye molecules exhibited comparable angle distributions against the host director and explored a full range of orientations around the director, as described in the Supporting Information (Figure S5 and S6). This simulation time is significantly longer than those reported for many liquid crystal MD studies, and it was required because of the small number of dye molecules in the simulation. During the simulations, small-scale motion about the equilibrium geometries was observed for both host and guest molecules, and no aggregation of the dye molecules was apparent. Figure 8 shows a snapshot of the final geometry of the simulation, at 500 ns.

**Figure 9:**
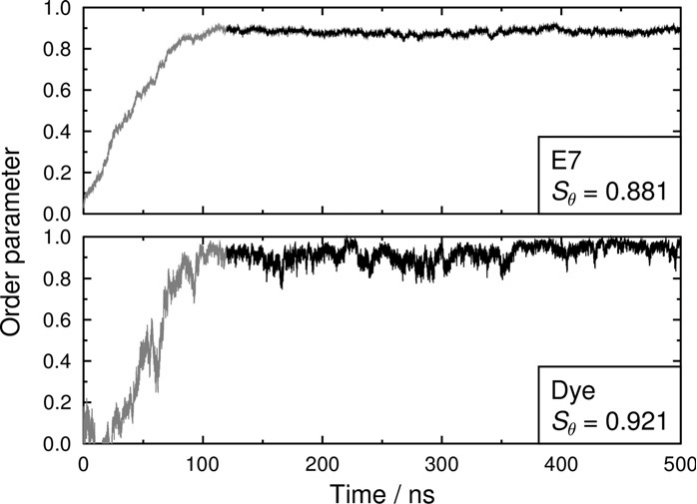
Order parameters of the E7 host molecules calculated by using Equation (5) (top), and of the 26B3OH dye molecules calculated by using Equation (2) versus the host director (bottom), averaged over all respective molecules for each time interval. The insets give order parameter values obtained by averaging over 120–500 ns, as shown by the ranges plotted in black.

An analysis of the alignment of the molecules during the guest–host simulation is shown by the order parameter plots in Figure 9. The guest–host system evolved to a nematic phase in approximately 120 ns, which is longer than the time of approximately 75 ns for the E7 host alone (described above). Averaging over 120–500 ns (the end of the run) gave an order parameter of *S*_*θ*_=0.881 for the E7 molecules in the guest–host system, matching that of *S*_*θ*_=0.885 obtained for the host alone (Figure S1 in the Supporting Information), and an order parameter of *S*_*θ*_=0.921 for the dye molecules versus the host director.

An additional guest–host simulation was run from a pseudo-nematic starting geometry, similar to that for the E7 host alone (described above). This simulation enabled averaging from an earlier time of 30 ns, and it gave order parameters for the E7 molecules (*S*_*θ*_=0.879) and dye molecules (*S*_*θ*_=0.896) that were comparable to those from the isotropic starting point (Figure S7 in the Supporting Information).

The order parameters from the simulations indicate that the dye molecules are more aligned than the host molecules in the guest–host system. They are also consistent with the higher experimental order parameter we observe for the dye in the guest–host system than that reported experimentally for the E7 host alone, both here and in the literature.[33] The effect of a guest dye having a higher order parameter than its host has been observed experimentally for a range of systems, and it generally arises for dyes that have slightly longer molecular structures than those of their hosts, as discussed in the literature.[6] Hence, the higher simulated and experimental order parameters obtained here for the 26B3OH dye than the E7 host may be attributable to its relatively long and rigid rod-like structure in comparison with the slightly shorter and more flexible host molecules (Figure 2).

The effect of adding the 26B3OH dye to the E7 host was also studied experimentally by measuring the nematic-isotropic transition temperature (*T*_NI_), which gave values of 59.4 °C for the host and 61.7 °C for the guest–host system. This observed increase in *T*_NI_ is attributable to the effect of adding a dye that exhibits liquid crystalline behaviour in its pure form and has a *T*_NI_ value of>390 °C,[35] which is much higher than that of E7. In general, the order parameter of a nematic system increases as the temperature is decreased at <*T*_NI_, which is often expressed in terms of the reduced temperature, *T*/*T*_NI_, at which an observation is made. In the system studied here, the increase of 2.3 °C in *T*_NI_ on adding the dye effectively lowers the reduced temperature of an observation made at 300 K from 0.902 to 0.896, which is a very small change that is unlikely to have a significant effect on the order parameter.

### Combining DFT and MD results

The separate calculations of the components of the order parameter of the dye, *S*_*β*_=0.999 from the TD-DFT calculation and *S*_*θ*_=0.921 from the MD simulation, combine using Equation (4) to give an overall value of *S*_*ϕ*_=0.920 for the dye in this guest–host system. The fact that this calculated value is higher than the experimental value of *S*_exptl_=0.74 is probably attributable, principally, to the MD simulations giving a host environment that is too highly ordered, as indicated by the high *S*_*θ*_ order parameters calculated for E7, as discussed above. The value of *S*_*β*_ obtained here from calculations on a static isolated molecule may also contribute to a high calculated value of *S*_*ϕ*_ because conformational changes may modify this value on going to a dynamic sample in the condensed phase. However, the calculated transition is mainly localised on the relatively rigid anthraquinone core of this symmetric dye, and such an effect is probably small for this particular system.

**Figure 10:**
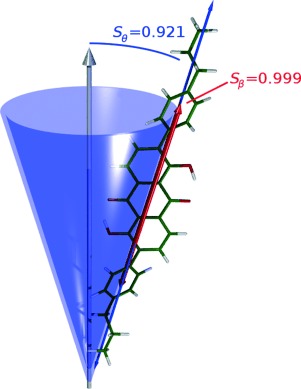
Schematic representation of the host E7 director and the calculated order parameters for the alignment of the 26B3OH dye and the TDM of its visible absorption transition. (In this pictorial representation: the cone is drawn at an angle of *θ*=13.3°, corresponding to that which would derive from applying Equation (2) to a hypothetical *δ*-distribution,[42] whereas the value of *S*_*θ*_ actually arises from a range of *θ* angles in the simulation, as shown in Figure S5 in the Supporting Information; the TDM is drawn at an angle of *β*=1.7° to the long axis of the dye.)

The calculated contributions of the components to the alignment of the 26B3OH dye within the E7 host are shown schematically in Figure 10. The calculated values indicate that the experimental dichroic ratio and associated order parameter of this particular dye in the E7 host are limited not by the alignment of the transition dipole with the long molecular axis of the dye (*S*_*β*_) but principally by the alignment of the dye within the host (*S*_*θ*_). This interpretation illustrates the value of expressing the overall order parameter of the dye as two separate components, and it provides a better understanding of the alignment of the guest–host system than given by the dichroic ratio measurement alone.

In a broader context, this study illustrates a general approach that uses a combination of DFT calculations, giving spectroscopic information, and MD simulations, providing dynamic information on molecular geometries, to develop an understanding of these contributions to the overall performance of a guest–host liquid crystal system, alongside experimental data. The computational approach described here was used without the input of experimental data, apart from default or literature values for the MD parameters, and, hence, it may offer the potential to assist in the rational design of new dyes and guest–host systems. Moreover, the MD simulations provide much more information than the second-rank order parameter <*P*_2_>, on which we have focused here, including full orientational distribution functions for the dye molecules and correlation functions for dye and E7 molecules, which may assist in the fundamental understanding of such guest–host systems.

## Conclusion

A guest anthraquinone dye has been shown experimentally to align within a nematic liquid crystal host. DFT calculations have given the alignment of the transition dipole moment within the dye, and fully atomistic MD simulations have modelled the alignment of the dye within the host. Importantly, the combination of DFT calculations and MD simulations presented here, along with experimental data on the same sample system, demonstrate a general approach to studying and understanding the alignment of guest molecules in liquid crystal hosts. This approach enables data from UV/Vis spectroscopic measurements on aligned samples to be compared directly with order parameters obtained from computational studies, and the DFT calculations also provide information on the nature of the transitions giving rise to the colour of the dye. This general approach may be applied readily to a range of different guest molecules and hosts.

In the specific example presented here, the modelled order parameters were higher than the experimental values, which may be attributable principally to limitations of the force field used in the MD simulations of the host. However, the ability to provide a quantitative prediction of guest alignment in a liquid crystal host without the input of experimental data offers the potential to compare different systems computationally, and, thereby, to provide a step towards an efficient method of rational design that may be relevant in developing new guest–host systems for display devices and other applications.

## Experimental Section

### Synthesis and experimental methods

The synthesis, purification and characterisation of 1,5-dihydroxy-2,6-bis-(4-propylphenyl)-9,10-anthraquinone (26B3OH) has been reported previously;[35] E7 (Merck) and *p*-xylene (>99 %; Sigma-Aldrich) were used as received. UV/Vis absorption spectra were recorded using a Hitachi U-3010 spectrophotometer. Solution samples were prepared by dissolving 26B3OH in *p*-xylene; spectra versus solvent were recorded at room temperature (ca. 298 K) using matched quartz cuvettes with a path length of 1 mm. Guest-host samples were prepared by heating a mixture of E7 and 26B3OH (ca. 1.5 wt %) above the clearing point to ensure full dissolution of the dye, and sonicating the mixture for about 2 min after cooling to room temperature; visual and microscope inspections of the mixture showed no evidence of precipitation. Cells for aligned samples were constructed by spin-coating glass microscope slides with a saturated solution of nylon-6,6 in formic acid, drying them in an oven at 100 °C, and then rubbing the nylon in a defined direction to provide alignment surfaces; two slides were used to make a cell with a path length of approximately 20 μm, and observation with a microscope and crossed polarizers confirmed the alignment of the guest–host samples. Polarized UV/Vis absorption spectra of aligned samples were recorded at 300 K versus air, using a Glan-laser polarizer (Newport 10GL08) between the lamp and the sample to polarize the beam. Initially, the sample cell was rotated to maximise the absorbance at the peak wavelength of the visible band and the parallel measurement was recorded; the cell was then rotated by 90° and the perpendicular measurement was recorded. The spectrum of an aligned sample of E7 alone was also recorded versus air at each of these orientations, and subtractions of these reference spectra were used to obtain the data presented. The N–I transition temperatures of E7 alone and of the guest–host system were obtained by microscopy, by using a Zeiss Axioskop 40 polarizing transmitted light microscope with a Mettler FP82HT microfurnace and FP90 central processor.

### Computational methods

DFT calculations were performed by using the Gaussian 09 software package.[43] The structural optimisation was carried out on an isolated 26B3OH molecule in an all-*trans* configuration by using the B3LYP functional[44,45] with the 6-31G(d) basis set. This optimised geometry was then used for the subsequent time-dependent DFT (TD-DFT) calculation, which was carried out at the same level of theory. A simulated UV/Vis absorption spectrum was generated by summing Gaussian bands (50 nm FWHM) with the peaks at the calculated transition wavelengths and with the respective oscillator strengths.

Fully atomistic MD simulations were carried out using the GROMACS 4.5.5 package.[46–49] The simulations used the OPLS AA force field[50,51] apart from the inter-ring torsions in 26B3OH and in molecules of the E7 mixture, for which reported biphenyl and cyanobiphenyl torsional force constants were used,[52] and the atomic charges within 26B3OH, for which HLY charges[53] calculated from the optimised DFT structure were used. Simulations were run by using 2 fs time-steps, periodic boundary conditions, and at 300 K and 1 bar maintained by using a velocity-rescale thermostat[54] and Parrinello–Rahman pressure coupling.[55] A van der Waals cut-off radius of 9 Å was used, and electrostatic interactions were calculated by using the Particle Mesh Ewald method with a cut-off of 9 Å.[56,57] All bond lengths were constrained throughout the simulation by using the P-LINCS algorithm.[58] Simulations of the E7 mixture were carried out with the component molecules in the relative wt % ratios given in Figure 2 (and with the numbers given in Table S1 in the Supporting Information); the sizes of the simulations are described in the main text, and further details of the MD simulation methods are given in the Supporting Information.

Minimum moment of inertia axes were defined as the eigenvectors associated with the minimum eigenvalues of the diagonalised moment of inertia tensors[59] calculated for structures obtained from both DFT and MD methods.
