# Supplementary material for: Dyes in Liquid Crystals: Experimental and Computational Studies of a Guest–Host System Based on a Combined DFT and MD Approach
Source: Chemistry. 2015 Jun 1;21(28):10123–30. doi: 10.1002/chem.201406372 (PMC4515093; doi:10.1002/chem.201406372)
Supplement: Supplementary file 1 [file chem0021-10123-sd1.pdf]

CHEMISTRY
A European Journal

Supporting Information

Dyes in Liquid Crystals: Experimental and Computational Studies of a Guest–Host System Based on a Combined DFT and MD Approach

Mark T. Sims, Laurence C. Abbott, Stephen J. Cowling, John W. Goodby, and John N. Moore*[a]

chem_201406372_sm_miscellaneous_information.pdf

CHEMISTRY
A European Journal

Supporting Information

Dyes in Liquid Crystals: Experimental and Computational Studies of a Guest–Host System Based on a Combined DFT and MD Approach

Mark T. Sims, Laurence C. Abbott, Stephen J. Cowling, John W. Goodby, and
John N. Moore*[a]

chem_201406372_sm_miscellaneous_information.pdf

General MD simulation methods

Molecular geometries from DFT optimisations (B3LYP functional; 6-31G(d) basis set) were used as the starting geometries for the MD simulations, for both host and dye molecules.

The MD studies were initiated by building a regular lattice of E7 component molecules randomly distributed in the correct ratios (Figure 2; Table S1), with their long axes aligned parallel to each other, with random head-tail alignment and no overall net head-tail alignment, and with the molecules regularly spaced by *ca.* 10 Å along each axis corresponding to a gas-phase density. An 8 × 8 × 4 lattice of 256 molecules was used for the 256-host molecule systems, whereas a 10 × 10 × 4 lattice was used for 400-host molecule systems, in each case giving an approximately cubic simulation box. For the guest-host systems, the five guest dye molecules were placed at random mid-positions between the host molecules, and with their long axes aligned parallel to those of the host molecules. Prior to running any simulations, an energy minimisation on the lattice systems was carried out using a steepest descent algorithm.

To obtain isotropic starting geometries for the MD simulations, an NVT run of 20 ps at a nominal temperature of 1000 K was carried out, enabling the lattice system to evolve into a randomised system for which the order parameter was ≈ 0, before rapidly compressing the system with a nominal pressure of 5×10^4 bar until reaching a density of ≈ 1 g cm^{-3}, corresponding to a condensed-phase, randomised system. The system was subsequently relaxed, again using a steepest descent method, before running the full MD simulations with periodic boundary conditions in all dimensions, and using 2 fs steps with the trajectory recorded at 10 ps time intervals. Pseudo-nematic starting geometries were obtained in the same way as the isotropic starting geometries but compressing the lattice system directly, without the initial, randomising NVT step.

Table S1. Numbers of component molecules used in the MD simulations with 256, 400 and 3872 total molecules of E7.

	Number of molecules		
E7 total	256	400	3872
5CB	141	220	2130
7CB	62	97	941
8OCB	36	56	542
5CT	17	27	259

MD simulations of E7 alone

Figures S1-S3 show results from the MD simulations of 256 molecules of E7 alone, using either isotropic or pseudo-nematic starting geometries (as indicated).

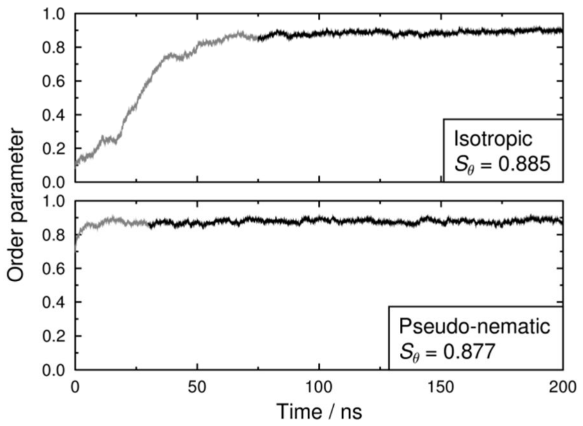

Figure S1. Order parameters, S_θ, calculated over all molecules at each time interval from the two simulations of 256 molecules of E7 alone, starting from isotropic (top) and pseudo-nematic (bottom) geometries. Insets give S_θ values obtained from averaging over 75-200 ns (top) and 30-200 ns (bottom), as shown by the ranges plotted in black.

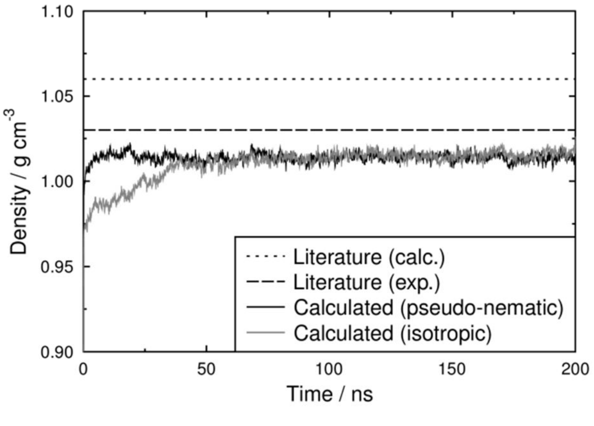

Figure S2. Densities were calculated at each time interval from the two simulations of 256 molecules of E7 alone, starting from isotropic (grey) and pseudo-nematic (black) geometries, and are plotted here as 10-point averages. Values from reported simulations[S1] and experimental measurement[S2] shown as dotted and dashed lines, respectively.

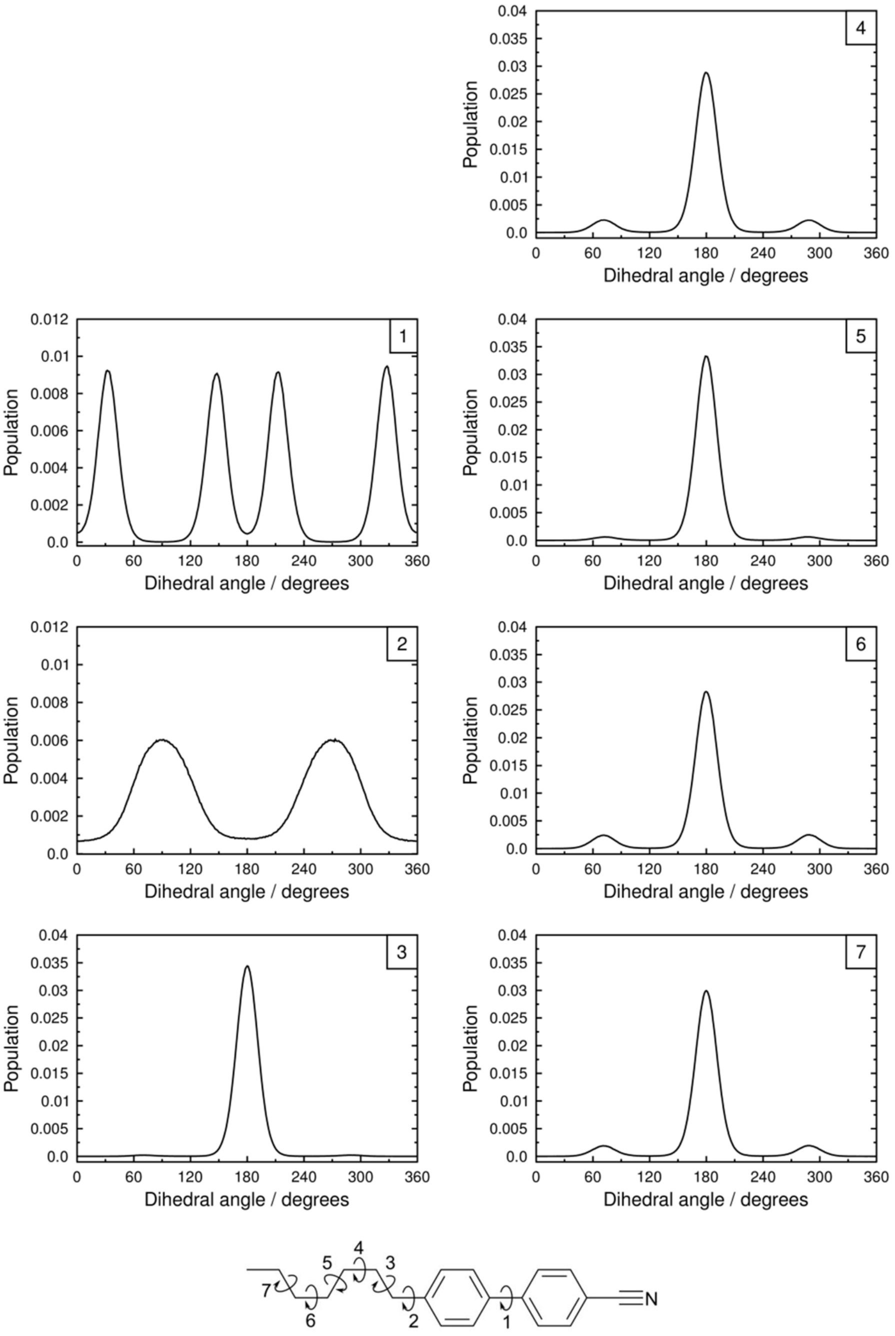

Figure S3. Normalised dihedral distribution functions of seven dihedral angles 1-7 in the E7 component molecule 7CB, as defined in the structure shown above, calculated from the 256-molecule simulation of E7 alone with a pseudo-nematic starting geometry, obtained between 30 ns and 200 ns. (*cf.* equivalent Figure 3 in reference S1).

Figure S4 shows results from an additional simulation of 3872 molecules of E7 alone, initially in a 8 × 22 × 22 lattice, which was run for 50 ns from a pseudo-nematic starting geometry and using the same parameters as the 256 molecule simulation. The calculated value of S_θ levelled off after 30 ns to give an average of 0.869 to the end of the run, which is comparable to the values calculated from the simulations reported here using fewer molecules of E7.

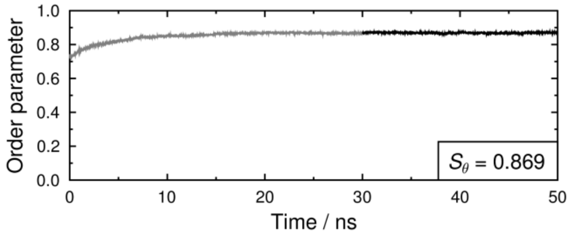

Figure S4. Order parameters, S_θ, calculated over all molecules at each time interval from the 3872-molecule simulation of E7 alone, starting from a pseudo-nematic geometry. The inset gives the S_θ value obtained from averaging over 30-50 ns, as shown by the range plotted in black.

Experimental order parameter of E7 alone

Electric permittivity measurements on E7 alone at 25 °C gave parallel and perpendicular electric permitivity values of $\varepsilon_{\parallel}$ = 18.8 and $\varepsilon_{\perp}$ = 5.5, respectively, which are comparable to values reported in the literature.[S3, S4] These values gave average and difference values of $\bar{\varepsilon}$ = 9.9 and $\Delta\varepsilon$ = 13.3, respectively, and Equation (S1)[S5] was used to give an estimated order parameter of S = 0.64 for E7. A comparable value of S = 0.65 for E7 is reported in the literature, from refractive index measurements.[S1]

$$S = \frac{\Delta\varepsilon}{3\left(\bar{\varepsilon} - 3\right)} \qquad \text{(S1)}$$

MD simulations of a guest-host mixture

Figures. S5 and S6 show angular distribution results from the MD simulations of 400 molecules of E7 and 5 molecules of 26B3OH, using an isotropic starting geometry.

Figure S5 shows histograms of the θ angles explored by each of the five dye molecules for three different time ranges, starting at 120 ns and ending at either 200, 300 or 500 ns. The shorter time range of 120-200 ns gives noticeably different histograms for the five dyes, with peak values in these distributions ranging from 16° (Dyes 2 and 4) to 6° (Dye 5), and average S_θ values ranging from 0.834 (Dye 2) to 0.954 (Dye 5). The results from the five dye molecules become more comparable with longer simulation times, as shown in Figure S5, and using a time range of 120-500 ns gives average S_θ values that are similar for all five dyes, with a much narrower range of 0.902-0.934. Hence, we considered 500 ns to be a suitable simulation time to study the five dye molecules in this guest-host mixture at an acceptable computational expense.

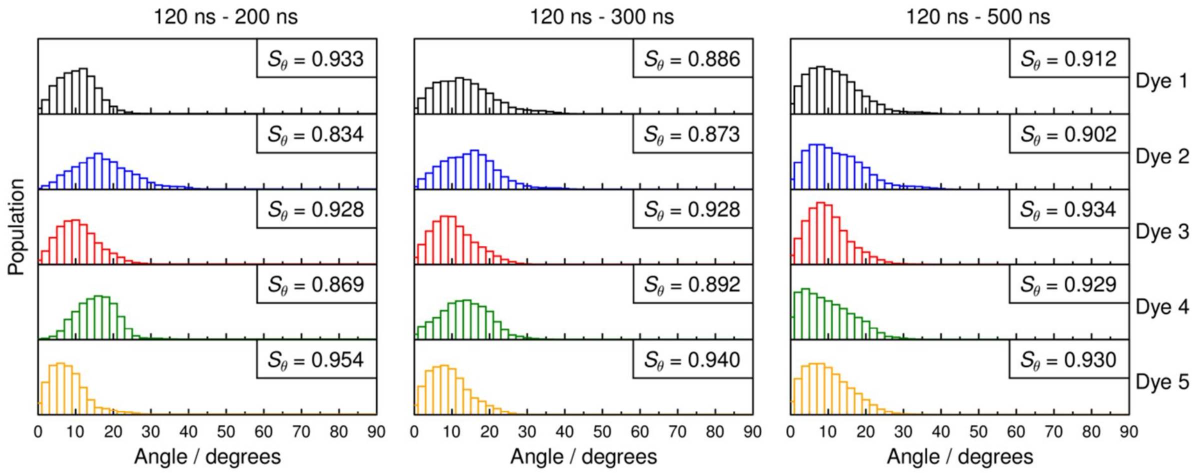

Figure S5. Histograms of the θ angles explored by each of the five 26B3OH molecules, Dye 1-5, during the guest-host simulation with an isotropic starting geometry; the angle was calculated between the director and the long molecular axis of each dye at each time interval. The histograms are plotted for ranges of 120-200 (left), 120-300 (middle) and 120-500 ns (right), and are normalised for each time range. Insets give S_θ values for each dye, 1-5, obtained by averaging over each time range.

Figure S6 shows unit vectors that estimate the orientations explored around the host director **n**, for each of the dye molecules and for the same three time ranges used in Figure S5. These plots are viewed down the host director axis, such that the vector directions in the plane of the page are perpendicular to the host director, estimating the orientations explored by the dye molecules around the blue cones shown schematically in Figures. 4 and 10, and the vector lengths in the plane of the page are projections determined by the θ angles (given in Figure S5).

The orientation of the director was not constrained in the MD simulation, in which the molecules were allowed to move freely, such that the system did not have a fixed reference/laboratory frame against which the director and perpendicular axes were defined. Hence, the plots in Figure S6 were generated by transforming the z-axis of the simulation box to align with the calculated host director **n** at each time interval, by sequential rotation about x and then y axes. Consequently, these plots show estimates rather than actual orientations explored around the director (whereas the θ angles given in Figure S5 are fully defined internally, without the need for a reference frame). When the system had evolved to a nematic phase at 120 ns, the calculated host director was found to remain in a relatively constant orientation within the axes of the simulation box, varying by $\approx \pm 5°$ during the time to the end of the run at 500 ns, and suggesting that this approach may provide reasonable estimates.

Despite the caveats given above, the plots in Figure S6 appear to provide a useful qualitative diagnostic tool to help in assessing possible limitations that might arise from studying a small number of guest molecules with a shorter simulation time than that used here. For example, Figure S6 shows that Dye 2 and especially Dye 4 appear to have explored only a relatively small range of angles around the host director in the time range of 120-200 ns, whereas they appear to have explored the full range of angles when the time range is extended to 120-500 ns. It is notable that Dyes 2 and 4 also gave low S_θ values in the time range 120-200 ns, and that these values increased to become comparable with those of the other dyes when the range was extended to 120-500 ns, as shown in Figure S5. Hence, Figure S6 provides additional qualitative support for the use of 500 ns as a suitable simulation time to study the five dye molecules in this guest-host mixture.

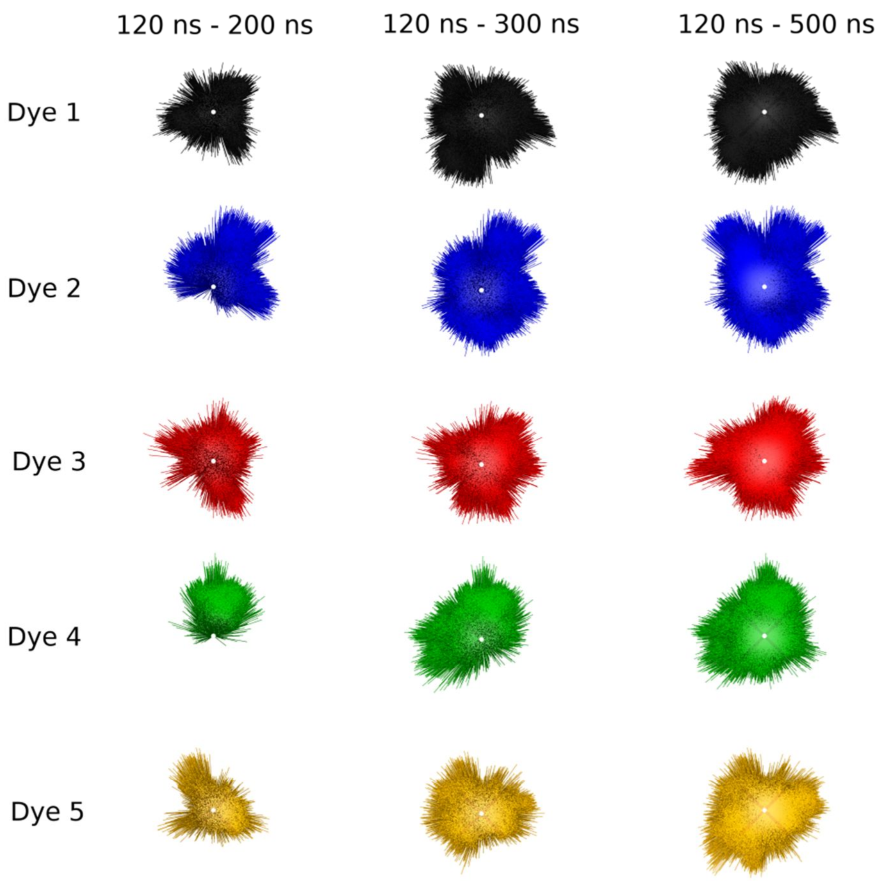

Figure S6. Estimates of the orientations explored by the long molecular axis of each of the five 26B3OH dye molecules, 1-5 (identities matching Figure S5), during the guest-host simulation with an isotropic starting geometry, drawn as unit vectors and viewed down the host director axis **n** (central point in white); each unit vector was calculated as the minimum moment of inertia axis of each dye at each time interval, and the plots show all the vectors for ranges of 120-200 (left), 120-300 (middle) and 120-500 ns (right).

Figure S7 shows results from the MD simulation of 400 molecules of E7 and 5 molecules of 26B3OH, using a pseudo-nematic starting geometries.

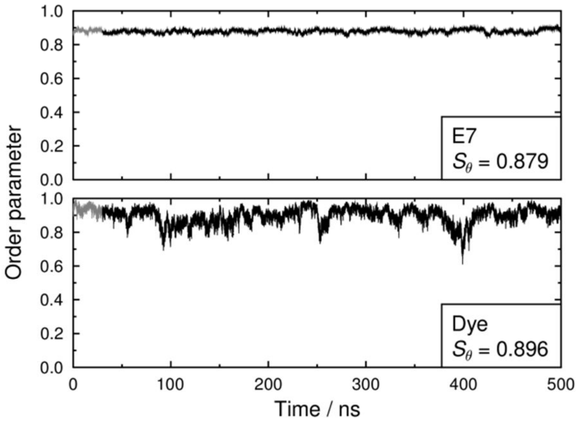

Figure S7. Order parameters, S_θ, calculated over all the E7 host molecules (top) and the five 26B3OH dye molecules (bottom) at each time interval from the guest-host simulation with a pseudo-nematic starting geometry. Insets give S_θ values obtained from averaging over 30-500 ns, as shown by the ranges plotted in black.

References

[S1] J. Peláez and M. Wilson, *Phys. Chem. Chem. Phys.*, **2007**, *9*, 2968-2975.

[S2] A. Y. G. Fuh, K. L. Huang, C. H. Lin, I.-I. C. Lin and I. M. Jiang, *Chin. J. Phys.*, **1990**, *28*, 551-557.

[S3] G. Y. Kim and C. H. Kwak, *Opt Commun*, **2011**, *284*, 5157-5163.

[S4] A. D. Remenyuk, E. V. Astrova, R. F. Vitman, T. S. Perova and V. A. Tolmachev, *Opto-Ireland 2005: Optoelectronics, Photonic Devices, and Optical Networks,* **2005**, *5825*, 400-407.

[S5] E. P. Raynes, personal communication.
